# Case from the Surgical Clinic of Prof. Moses Gunn, of Rush Medical College

**Published:** 1870-11

**Authors:** H. F. Chesbrough


ARTICLE III. — *Case from the Surgical Clinic of Professor Moses Gunn, of Rush Medical College.* Reported by H. F. CHESBROUGH, M.D.

[Continued.]

IX. On Saturday, May 21, S. A. H., of Wisconsin, presented a letter from Dr. H. C. Soule. He had come for relief from a tumor in the fauces. It was with great difficulty that he could make himself understood, the tumor presenting an almost total impediment to speech. He presented the appearance, every time he moved a few steps, of a man laboring under a severe attack of spasmodic asthma. His color was bad, face care-worn, figure stooping, and whole appearance that of a man who had long struggled against an incurable disease. Twelve years ago he had first noticed the tumor in the right side of the velum pendulum palati. At first it was of slow growth, gave very little trouble, and was not at all painful. But about three years ago the inconvenience became very great, so that he was forced to abstain from work entirely, because the size of the tumor prevented the ingress of sufficient air into the lungs to allow of any exertion. Two years ago it began to interfere with his sleep, so that at last he could, according to his own account, only sleep for about two minutes at a time. The tumor would fall upon the rima glottidis, and, acting as a valve, cut off the supply of air; then he would awake, and this process would be repeated till the night was passed. Of course through these last years his food had been restricted to liquids. On requesting the patient to open his mouth, there could be seen a large dark red mass seemingly filling up almost the entire cavity of the mouth, pharynx and fauces. By pressing the tumor towards the right with the finger, the uvula could be seen far back and to the left, crowded against the wall of the pharynx. Exploration with the finger also determined that the attachments of the tumor corresponded exactly with those of the velum pendulum palati. On the left side the index could be swept along the lower free border of the tumor, but on the right side the finger failed to quite reach the extremity of the recess. The tactile sense showed it to be solid and elastic. The exploring needle brought blood only—but blood very freely. The mucous membrane covering it was very red and vascular, so that, judging from the appearance, the situation, and the free hæmorrhage, the diagnosis of soft cancer seemed very probable. On the other hand, the absence of pain, the long history, the slow growth and non-ulceration of the tumor, pointed equally as strongly in the opposite direction.

The diagnosis, therefore, was doubtful, with an inclination toward that of a benignant tumor.

The patient was informed of his danger. He was told that he would undoubtedly be suffocated by the tumor at no distant day if it was left untouched; that the operation might be difficult, dangerous and even fatal, but, if successful, would yield partial, and possibly complete relief. He was given the plain choice between letting the tumor alone, and certain death in the not-far-distant future on the one hand, and a dangerous operation with hope of great relief on the other. He took time to consider, and on Monday morning announced his intention of undergoing the operation. (He afterwards said that he came to Chicago with the intention of having the tumor removed at all hazards.) Tuesday, 2 P. M., was the time fixed upon. The patient was punctual, and

Dr. Gunn had provided himself with a charcoal furnace and three irons for the actual cautery. Dr. W. C. Hunt was also present, by invitation, with his microscope. An incision was first made into the substance of the tumor, and a portion of it removed and placed upon the slide of the microscope. Dr. Hunt immediately pronounced it fibrous. Dr. Gunn then commenced the operation with a curved, probe-pointed bistoury, sweeping from left to right close to and along the free border of the hard palate. He then endeavored to enucleate the tumor, and to his great satisfaction found this perfectly feasible. The patient was sitting in a chair facing the class, an assistant holding his head. He had so little space for breathing, that the additional room taken by the surgeon's finger completely cut off his allowance of air. In a few moments he threw up his arms as a signal of distress, and was given a short respite. A second time the operation of enucleation was continued, and a second time the patient was allowed a chance to rest and recover breath. A third trial now peeled off the greater part of the mucous membrane from the tumor, but when the surgeon withdrew the finger, the patient still threw his hands wildly about and rolled his head from side to side without drawing a single breath. Before the class had time to comprehend the difficulty, the operator plunged his index and middle fingers into the patient's mouth and lifted the tumor from its valve-like position on the rima glottidis, pushing the mass upward and backward with sufficient force to leave room for the admittance of air. After another short breathing space had been given, Dr. Gunn swept his finger swiftly around the remaining attachments of the tumor, and amidst the applause of the spectators, dragged out of the patient's mouth a mass whose size taxes severely the imagination to understand how it could have rested so long in its place. Its greatest length was 3½ in., breadth 3 in., and thickness 2¼ in.

The mucous membrane peeled from the tumor was very dark and livid, and seemed almost certain to slough off, but the next day, on the contrary, it showed evident signs of contracting and effecting a restoration of the velum palati. An abscess formed within the pharynx and broke in the course of the week, and still the two folds of mucous membrane kept continually closing upon each other. It was supposed that it would be necessary eventually to put a stitch in, to close completely the gap, so when the patient went home at the end of two weeks he was told to return in six weeks for any operation which should then be indicated. At the end of that time he came back, his speech good, though not perfect by any means, the velum pendulum palati completely re-united, and the uvula in its natural position. He said he slept and ate perfectly well, and had gone to work soon after returning home, greatly to the astonishment of his family and friends, who had never expected to see him return alive.

There are a few points of especial interest in this case:

1. The situation of the tumor, no mention being made (as far as known) of a fibrous tumor in the vail of the palate.

2. The complete restoration of the velum pendulum palati.

3. The fact that the tumor was developed entirely in the right half of the velum (as proved by the integrity and position of the uvula) adds still more to the wonder at the restoration of the palate.

